# *Lacticaseibacillus paracasei* AD22 Stress Response in Brined White Cheese Matrix: In Vitro Probiotic Profiles and Molecular Characterization

**DOI:** 10.1007/s12602-024-10216-4

**Published:** 2024-02-29

**Authors:** Adalet DISHAN, Zafer GÖNÜLALAN

**Affiliations:** 1 Faculty of Veterinary Medicine, Dept. of Food Hygiene and Technology, Yozgat Bozok University, Yozgat, Türkiye; 2 Faculty of Veterinary Medicine, Dept. of Veterinary Public Health, Erciyes University, Kayseri, Türkiye

**Keywords:** Cheese, *L. paracasei*, Probiotic, Stress factors, Whole genome sequence

## Abstract

Functionalizing foods involve discovering and integrating new candidate health-promoting bacteria into the food matrix. This study aimed (i) to reveal the probiotic potential of autochthonous *Lacticaseibacillus paracasei* AD22 by a series of in vitro tests and molecular characterization and (ii) to evaluate its application to the matrix of brined white cheese, which is the most common cheese in Türkiye, in terms of survival and stress response. To evaluate in vitro probiotic characteristics, *L. paracasei* AD22 was exposed to functional, technological, and safety tests. Pilot scale production was conducted to integrate *L. paracasei* AD22 into the brined white cheese matrix. The expression levels of stress-related genes (*dnaK*, *groES*, *ftsH*, *argH*, and *hsp20*) were detected by reverse-transcriptase polymerase chain reaction to determine the transcriptional stress response during ripening. The presence of genes encoding stress-related proteins was determined by whole-genome sequence analysis using a subsystem approach; the presence of antibiotic resistance and virulence genes was determined by ResFinder4.1 and VirulenceFinder 2.0 databases. The BAGEL4 database determined the presence of bacteriocin clusters. *L. paracasei* AD22 was found to survive in pH 2 and medium with 12% NaCl and did not cause hemolysis. Adhesion of the strain to Caco2 cells was 76.26 ± 4.81% and it had coaggregation/autoaggregation properties. It was determined that *L. paracasei* AD22 exceeded 7 log cfu/g in the cheese matrix at the end of the ripening period. Total mesophilic aerobes decreased in the cheese inoculated with *L. paracasei* AD22 after the 45th day of ripening. While *hsp20* and *groES* genes were downregulated during ripening, *argH* was upregulated. Both downregulation and upregulation were observed in *dnaK* and *ftsH*. Fold changes indicating the expression levels of *dnaK*, *groES*, *ftsH*, *argH*, and *hsp20* genes were not statistically significant during ripening (*p* > 0.05). Whole-genome sequence profiles revealed that the strain did not contain antibiotic and virulence genes but bacteriocin clusters encoding Enterolysin A (Class III bacteriocin), Carnosine CP52 (class II bacteriocin), Enterocin X beta chain (Class IIc bacteriocin), and the LanT region. Subsystems approach manifested that the most functional part of the genomic distribution belonged to metabolism, protein processing, and stress response functions. The study findings highlight that *L. paracasei* AD22 will provide biotechnological innovation as a probiotic adjunct because it contains tolerance factors and probiotic characteristics to produce new functional foods.

## Introduction

Recent scientific research has focused on selecting and characterizing new species and more specific probiotic strains that survive in the food matrix and contain functional properties [1, 2]. In particular, the isolation of new probiotic strains from traditional food products such as kefir grains, cheese, fermented olives, meat products, and the possible usage of new isolates in food production has been taken into consideration [3–7]. Probiotics are part of functional foods and are proposed as one of the main mechanisms providing beneficial effects such as protecting mucosal barrier, preventing the proliferation of pathogens, synthesizing immunomodulatory the cellular metabolites in the host [8].

Viability in the product matrix during food production, distribution, and storage is a factor assigning the efficacy of probiotics and starter cultures. Cheese is described as a suitable food matrix for the delivery of probiotics; hence, studies have emphasized the positive effect of probiotic addition on the physicochemical, microbiological, and sensory properties of cheese [9, 10]. Cheese is a decent option for probiotics with advantages in terms of better buffering capacity, less water activity, and low storage temperature over other fermented milk products [11]. However, a strain designated as a starter culture, probiotic, or adjunct culture must be present in sufficient quantities to meet industrial requirements. High survival stability in the cheese production process and before inclusion is important in determining probiotic properties [11]. Therefore, it is essential to reveal the stress response of lactic acid bacteria (LAB) and the molecular regulatory mechanism of the process under environmental stresses to reduce stress-related damage and increase the survival rate of the product.

Research on strains used as probiotics is generally predicated on functional properties, but information on their stress tolerance capacity integrated into food matrix and storage is insufficient. Model food systems are required since when a new strain is incorporated into the cheese production process, it must exhibit appropriate survival behavior and control its effects on carbohydrate, protein, and fat utilization [12].

In this study, the following were aimed: (i) to reveal the probiotic potential of autochthonous *L. paracasei* AD22 by a series of in vitro tests and molecular characterization and (ii) to evaluate its integration into the matrix of brined white cheese in terms of survival and stress response.

## Materials and Methods

### Isolation and Identification of *L. paracasei* AD22

In this experimental study, cheese samples (*n*: 25) were obtained from the villages of Kayseri province between November and December 2021, which were produced from raw milk and stored in plastic barrels for at least six months to mature. The cheese samples (25 g) were kept anaerobically at 37 °C in 225 mL Man, Rogosa, and Sharpe (MRS; Merck, Germany) broth for overnight incubation. Each inoculum (100 μL) was spread on MRS agar and anaerobically incubated at 37 °C for 48 h [13]. Selected colonies were purified and examined according to gram staining and catalase test. The tolerance properties of the selected isolates were examined and the genomic DNAs (gDNA) of isolates were subjected to 16S rRNA sequence analysis. For identification of L. paracasei AD22, gDNA was amplified by Polymerase Chain Reaction (PCR) using universal primers targeting 16S rRNA gene region (27F:5′-AGAGTTTGATCCTGGCTCAG -3′, 1492R: 5′- GGTTACCTTGTTACGACTT -3′) [14]. The amplicons were subjected to Sanger sequencing by Macrogen (South Korea). Paired nucleotide sequences were assembled using the program Geneious Prime 2020.1 (https://www.geneious.com). It was decided to use *L. paracasei* AD22 for the pilot scale application and characterize it molecularly. Stock cultures of the isolates are kept at – 80 °C in MRS medium with glycerol (20% v/v) in the culture collection of Food Hygiene and Technology Department Laboratuary, Faculty of Veterinary Medicine.

### In Vitro Physiological and Safety Traits of *L. paracasei* AD22

pH tolerance (pH 2, 3 and 7), bile salt tolerance (0.3% and 0.6% bile of bovine origin) and the simulated gastric juice tolerance (%0.3 pepsin at pH 2.5) tests applied by Zheng et al. and the simulated intestinal juice tolerance (adjusting to pH 8.0 with 1 N NaOH by containing 1 mg/L pancreatin, 0.5%NaCl) tests applied by de Oliveira et al. were followed with slight alterations. pH, simulated gastric and intestinal juice tolerance were tested at the 3rd hour of incubation; bile tolerance was analyzed at the 3rd and 24th hour of incubation [15, 16]. Plate counting assessed cell viability and results were expressed as log cfu/mL. NaCI tolerance was evaluated according to Optical Density (OD) indicating turbidity formation measured at 600 nm (OD: 0.10–0.30 indicated slightly turbid, positive poor growth; OD:0.30–0.50 indicated medium turbidity, good growth; OD > 0.60 indicated very turbid, very good improvement; OD < 0.10 indicated negative growth) [17]. Determination of autoaggregation and cell surface hydrophobicity properties was performed via the method described by Yasmin et al. with minor modifications [18]. A coaggregation test was conducted against *Staphylococcus aureus* ATCC 25923, *Salmonella* Typhimurium NCTC 74, *Escherichia coli* ATCC 25922, and *Listeria monocytogenes* N7144 by adjusting the bacterial suspension of pathogenic bacteria and L. paracasei AD22 at OD600 to 0.5 ± 0.02 [19]. Bacterial adhesion to Caco2 cells was applied according to the method specified by Xu et al. [20]. Antibacterial activity of cell-free supernatant (CFS, 80 μL) from *L. paracasei* AD22 against *Escherichia coli* ATCC 25922, *Salmonella* Typhimurium NCTC74, *Listeria monocytogenes* N7144, and *Staphylococcus aureus* ATCC 25923 adjusted to 0.5 MacFarland turbidity was determined by the agar well diffusion method [19]. CFS was obtained by filtering the supernatant after centrifuging an overnight culture inoculated into MRS broths [21]. Antibacterial activity was also determined against selected pathogens using the spot-on lawn method [22]. Antibiotic susceptibility of *L. paracasei* AD22 to selected antibiotics [vancomycin (VA, 30 μg; Oxoid, UK), trimethoprim and sulfamethoxazole (SXT, 25 μg; Bioanalyse, Türkiye), ampicillin (AMP, 10 mcg; Bioanalyse, Türkiye), cefotaxime (CTX), 30 mcg; Bioanalyse, Türkiye), clindamycin (DA, 2 μg; Bioanalyse, Türkiye), tetracycline (TE, 30 μg; Bioanalyse, Türkiye), streptomycin (S, 10 mcg; Bioanalyse, Türkiye), ciprofloxacin (CIP, 5 μg; Bioanalyse, Türkiye), and erythromycin (E, 15 μg; Oxoid, UK)] were analyzed by disc diffusion method [23]. Inhibition zone diameters of ≤ 14 mm, ≥ 20 mm, and 15–19 mm were considered as resistant (R), sensitive (S), and intermediate (I), respectively [24]. Hemolytic activity was determined using blood agar containing 5% (w/v) sheep blood and plates were incubated at 37 °C for 48 h. Hemolysis status was classified according to the lysis of red blood cells in the medium around the colonies. Green regions around colonies (α-hemolysis), clear regions around colonies (β-hemolysis), and no regions around colonies (γ-hemolysis) indicated hemolysis status. *S. aureus* ATCC 25923 strain was used as positive control. *L. paracasei* AD22 was tested for its proteolytic activity on skimmed milk agar medium. A clear or opaque region surrounding the wells indicated positive protease activity [25].

### Pilot Scale Cheese Production

Raw milk (10 L) was obtained thrice from Jersey dairy cows in a local farm. Pilot-scale experimental brined white cheeses were grouped as cheese containing *L. paracasei* AD22 and culture-free control cheese. After pasteurization (72 °C for 20 s), the milk (10 L) was cooled at 42 °C. Calcium chloride solution (0.2% w/v) was added to the cheese milk, followed by *L. paracasei* AD22. The culture grown overnight on MRS agar was washed three times with 0.5% saline by centrifugation (4000 rpm, 10 min). The suspension (50 mL), adjusted to 1 (5x10^8^ cfu/mL) with 0.5% saline at OD600, was added to 10 L of milk. Rennet (2 mL/100 L) was added to the milk (pH 6.6) when the milk was at 37 °C and coagulation took place in 90 min. Following coagulation, the clot was cut into cubes (3 to 4 cm^3^) and rested for 10 min. After leaching of the whey (without pressing), pressure was applied at room temperature for eight hours or until the whey drainage stopped. Next, the weights were removed, and the cheese block cut into cubes (3 to 4 cm^3^) was placed in autoclaved brine (12% NaCl). Precautions were taken to prevent cross-contamination at all stages of cheese making and the glass jars were autoclaved. Cheese groups were ripened for 60 days at 4 °C.

### Total Mesophilic Aerobes and Lactobacilli Counting

Microbiological cultivations were carried out at certain intervals (days 0, 1, 3, 7, 15, 30, 45, and 60) during the ripening of the cheeses. To count the total aerobic mesophilic microorganisms on each test day, serial dilutions were prepared with sterile phosphate buffered saline (1:9, pH 7.3) and inoculated onto Plate Count Agar (PCA; Merck, Germany) and incubated at 30 °C for 48–72 h. To count lactobacilli, serial dilutions were inoculated onto MRS agar (Merck, Germany) and incubated at 37 °C for 48–72 h. Analyses were carried out in two parallels. All cell numbers were expressed as logarithms of average colony-forming units per gram of cheese [10].

### RNA Extraction, cDNA Synthesis, and Reverse Transcriptase PCR

RNA extraction and determination of RNA concentration (ng/µL), cDNA synthesis and RT-PCR reaction were implemented according to the manufacturer's instructions for the kits mentioned hereinafter. RNA extraction from cheese was performed with the TransZol Up RNA extraction kit (TransGen Biotech, China). The amount of RNA was determined with the Qubit RNA HS Assay Kit (Thermo Fisher Scientific, USA). cDNA was synthesized from two separate RNA extractions concentration adjusted to 30 ng/µL. A high-capacity cDNA Reverse Transcription Kit (Thermo Fisher Scientific, USA) was used for cDNA synthesis. For each reaction, adjusted concentration of 10 μL RNA was added to the mixture containing 10X RT Buffer (2.0 μL), 25X dNTP Mix (100 mM, 0.8 μL), 10X RT Random Primers (2.0 μL), MultiScribeTM Reverse Transcriptase (1 μL), Nuclease-free H2O (4.2 μL). Reverse Transcriptase PCR reaction mixture containing 3.5 µL iQ™ SYBR® Green Supermix (BioRad, USA), 0.5 µL cDNA template, 0.5 µL forward primer (10 µM), 0.5 µL reverse primer (10 µM), and 5 µL sterile water the mixture with a volume of 10 µL was prepared. Each analysis was run with a DNA-free control. Livak and Schmittgen’s 2^−ΔΔCT^ calculation method was used to determine stress-related gene expression level changes [26]. Primer sequences are given in Table 1.

**Table 1:** Primer sequences for PCR amplification

Target gene	Nucleotide sequence	Reference
*argH*-F	GCAACGGAGTTAGCGGATTA	This study
*argH*-R	TTCCTGCAAGGCAGTGTT	
*ftsH*-F	TGCGCGATAATGGGACAA	
*ftsH-*R	CCAGATGGCTGGAGTGAATAA	
*dnaK*-F	CCGTTGTCTCTTGGGATTGA	
*dnaK*-R	CAACGGCTGGTTGACTATCT	
*hsp20*-F	ATGTGGATGTACCTGGGATTG	
*hsp20*-R	GGTCGGTGATATCGTCCTTATG	
*groES*-F	GTTGAAGAAGAGGAGCAGACAG	
*groES*-R	CCTTCACCTACTGCAACAACT	
16S-F	GAAGAATGGTCGGCAGAGTAA	
16S-R	CGCTTGCCACCTACGTATTA	

### Whole-Genome Sequencing

#### DNA Extraction and Fragment Analysis

Genomic DNA (gDNA) of *Lacticaseibacillus paracasei* AD22 was extracted using the Pure Link Genomic DNA Mini Kit (Invitrogen, USA) according to the manufacturer’s instructions. The concentrations of gDNA from the samples and the level of integrity and purity were checked using the Agilent 5400 Fragment Analyzer system. The obtained gDNA was subjected to DNA library preparation using Nextera XT DNA Library Preparation Kit (Illumina, USA) before being transferred to the next-generation sequencing platform. The components other than the fragments to be sequenced in the library were removed following the AMPure XP Bead (Beckman Coulter, UK) kit manufacturer’s instructions. The sequencing process was performed using the NovaSeq 6000 platform (Illumina, USA) with 2 × 150 bp double-ended (PE) chemistry. At the end of the sequencing process, the whole-genome data of the samples were obtained in the “fastq.gz” format.

### Bioinformatic Analysis

The Trim Galore open-access program was used to trim adapters and barcodes in the raw readings obtained [27]. In the trimming stage, the -q 10 argument was set to have a base quality score of 10. De novo assembly analyses trimmed.fastq format reads with SPAdes 3.13.0 [28]. Mapping of the isolate with the reference genome was performed with the ProgressiveMauve open-access program using the *Lacticaseibacillus paracasei* ATCC 27092 (GenBank accession number: NZ_JAMOIQ000000000.1) record as the reference genome [29]. The RAST algorithm was used in the annotation of the strain [30]. The web-based Proksee program was used for the circular graphic representation of the genome assembly and annotation distribution (https://proksee.ca/). Functional subsystems approach was used for the functional genomic distribution of the strain [31]. The BAGEL4 open-access database was used to reveal the bacteriocin clusters [32]. ResFinder4.1 and VirulenceFinder 2.0 databases were used to detect the presence of antibiotic resistance and virulence genes [33, 34].

### Statistical Analysis

The statistical significance of the effects of different concentrations and times on the survival profiles of *Lacticaseibacillus paracasei* AD22 in tested environments was determined by analysis of variance. The effect of ripening time on total mesophilic aerobe count, lactobacilli count, and pH of cheeses was determined by analysis of variance. Additionally, the statistical significance of the difference in total mesophilic aerobe count, lactobacilli count, and pH change between the control and *L. paracasei* AD22 inoculated cheese groups was determined by analysis of variance. The effect of ripening time on the expression levels of stress-related genes was also examined by analysis of variance. Post hoc analysis was applied to compare intragroup results in each test (Duncan’s multiple comparison, *p* < 0.05). All statistical analyses were performed using the SPSS 24.0 statistical program [35].

## Results

The sequence obtained from the assembly data belonged to 16S rRNA sequencing analysis of the gDNA of *Lacticaseibacillus paracasei* AD22 has been deposited in GenBank under accession number OR143727.

### In Vitro Characterization

#### Tolerance Characteristics

Tolerance to pH, bile salt, simulated gastric juice, and simulated intestinal juice are shown in Table 2. The effect of different pH exposures on the survival of *L. paracasei* AD22 was found to be statistically significant (*p* < 0.05). The effect of different bile salt concentration on the survival rate of *L. paracasei* AD22 at 3 h was not significant (*p* > 0.05), but significant at 24 h (*p* < 0.05). Absorbance values measured at OD600 nm as a result of exposure to environments containing 4%, 8%, and 12% NaCl are shown in Table 2. While moderate turbidity and good growth were detected after exposure to salt-free medium and 4% NaCl medium, less turbidity and weak growth were observed in 8% and 12% NaCl medium. The effect of different salt concentrations on the viability of *L. paracasei* AD22 was found to be significant (*p* < 0.05). However, the effect of salt-free medium and 4% NaCl medium on the survival rate was not statistically different from each other (*p* > 0.05). The effect of 8% and 12% NaCl environments on the survival rate was also statistically insignificant from each other (*p* > 0.05).

**Table 2:** Tolerance characteristics of *L. paracasei* AD22

pH tolerance	3rd hour	
pH 2	31.00^c^ ± 0.53	
pH 3	100.29^a^ ± 0.16	
pH 7	94.35^b^ ± 0.88	
Simulated gastric juice tolerance	96.95 ± 1.66	
Simulated intestinal juice tolerance	99.80 ± 0.81	
Bile salt tolerance	3rd hour	24th hour
0.3%	95.27 ± 1.59^A^	65.80 ± 0.85^Bb^
0.6%	96.58 ± 1.25^A^	78.75 ± 0.71^Ba^
NaCl tolerance (OD 600 abs)	24th hour	
0%	0.448 ± 0.02^a^	
4%	0.438 ± 0.02^a^	
8%	0.148 ± 0.02^b^	
12%	0.140 ± 0.007^b^	

^A, B^Means shown with different exponential letters in the same row are statistically different (*p* < 0.05)

^a, b, c^Means shown with different exponential letters in the same column are statistically different (*p* < 0.05) (statistical analysis of this table was evaluated for each test)

### Cell Surface Hydrophobicity, Autoaggregation, Coaggregation, and Bacterial Adhesion to Caco2 Cells

Cell surface hydrophobicity and autoaggregation values were given in Table 3. The effect of time change on the level of autoaggregation was found to be significant (*p* < 0.05). Coaggregation potential against *Staphylococcus aureus* ATCC 25923, *Salmonella* Typhimirium NCTC 74, *Escherichia coli* ATCC 25922, and *Listeria monocytogenes* N7144 was given in Table 3. The coaggregation values obtained against each standard pathogen at the 2nd, 20th, and 24th hours were statistically significant (*p* < 0.05). When standard pathogens were compared, the coaggregation values obtained at each tested time were statistically different (*p* < 0.05), except for values against *Escherichia coli* ATCC 25922 and *Listeria monocytogenes* N7144 at the 24th hour. The adhesion percentage of *L. paracasei* AD22 to Caco2 cells is given in Table 3.

**Table 3:** Hydrophobicity, autoaggregation, in vitro adhesion, and coaggregation of *L. paracasei* AD22

Hydrophobicity	2nd hour				
	28.73 ± 1.25				
Autoaggregation	2nd hour	20th hour			24th hour
	18.21 ± 0.66^C^	58.87 ± 2.05^B^			65.86 ± 2.36^A^
Caco2 adhesion	2nd hour				
	76.26 ± 4.81				
Coaggregation	2nd hour		20th hour	24th hour	
*S.* Typhimirium NCTC 74	10.51 ± 0.22^Bc^		60.91 ± 0.06^Ac^	61.11 ± 0.03^Ab^	
*E. coli* ATCC 25922	17.89 ± 0.24^Ca^		63.36 ± 0.10^Bb^	67.78 ± 0.39^Aa^	
*L. monocytogenes* N7144	11.57 ± 0.06^Cb^		65.49 ± 0.09^Ba^	67.56 ± 0.10^Aa^	
*S. aureus* ATCC 25923	9.52 ± 0.02^Cd^		53.83 ± 0.45^Bd^	58.07 ± 0.02^Ac^	

^A, B, C^Means shown with different exponential letters in the same row are statistically different (*p* < 0.05)

^a, b, c, d^Means shown with different exponential letters in the same column are statistically different (*p* < 0.05) (statistical analysis of this table was evaluated for each test)

### Antimicrobial Activity

The inhibition effect of *L. paracasei* AD22 on *E. coli* ATCC 25922, *S*. Typhimurium NCTC 74, *S. aureus* ATCC 25923, and *L. monocytogenes* ATCC N7144 were determined as 13.5 ± 0.7 mm, 14.5 ± 0.7 mm, 11 ± 0.3 mm, and 13.5 ± 0.7 mm, respectively. Also, according to the spot-on lawn test, zone formation was observed against each standard pathogen examined.

### Antibiotic Susceptibility

The sensitivity of *L. paracasei* AD22 against antibiotics is indicated in Table 4.

**Table 4:** Antibiotic resistance profile of *L. paracasei* AD22

Antibiotics	Antibiotic group	Zone (mm)	Profile
CTX	Beta lactam	35.5 ± 0.7	S
AMP	Penicillin	33.5 ± 0.7	S
DA	Lincosamide	51 ± 1.4	S
SXT	Sulfonamide	21.5 ± 0.7	S
E	Macrolide	40.5 ± 0.7	S
TE	Tetracycline	52.5 ± 0.7	S
CIP	Fluoroquinolone	13.5 ± 0.7	R
VA	Glycopeptide	-	R
S	Aminoglycoside	-	R

*S* susceptible, *R* resistant, *I* intermediate

### Hemolytic and Proteolytic Activity

As a result of the hemolytic activity test, it was observed that *L. paracasei* AD22 did not form a zone. On skim milk agar medium, *L. paracasei* AD22 formed zone indicating the proteolytic activity.

### Microbiological Enumeration and pH Change

The total mesophilic aerobe and lactobacilli counts of samples on the 1st, 3rd, 7th, 30th, 45th, and 60th days of the ripening period are given in Table 5. There is a statistically significant difference among the groups in the total aerobic mesophilic microorganisms and the lactobacilli counts (log cfu/g) throughout ripening (*p* < 0.05). A decrease in total aerobic mesophilic microorganisms was observed in the cheese inoculated with *L. paracasei* AD22 after the 45th day. The pH values measured on the 1st, 3rd, 7th, 30th, 45th, and 60th days of the ripening period are also given in Table 5. The pH change between the groups was statistically significant (*p* < 0.05) from the first day.

**Table 5:** Total aerobic mesophilic microorganism (log cfu/g), lactobacilli count (log cfu/g), and pH change of cheese groups

	PCA		MRSA		pH change	
Time	Control	AD22	Control	AD22	Control	AD22
Curd	6.67 ± 0.02^abc^	6.49 ± 0.08^a^	2.31 ± 0.06^Bc^	6.42 ± 0.34^Ac^	6.46 ± 0.01^a^	6.45 ± 0.01^a^
1st day	5.40 ± 0.12^Ad^	5.34 ± 0.06^Bc^	2.25 ± 0.04^Bbc^	6.59 ± 0.22^Abc^	6.41 ± 0.05^Aab^	6.32 ± 0.01^Bb^
3rd day	6.07 ± 0.89^Ac^	5.81 ± 0.08^Bb^	2.42 ± 0.16^Bbc^	6.58 ± 0.15^Abc^	6.36 ± 0.04^Aabc^	6.13 ± 0.03^Bc^
7th day	6.25 ± 0.23^Abc^	6.29 ± 0.10^Ba^	2.48 ± 0.01^Bbc^	6.58 ± 0.22^Abc^	6.34 ± 0.02^Aabc^	6.00 ± 0.1^Bd^
15th day	6.42 ± 0.49^Aabc^	6.38 ± 0.09^Ba^	2.54 ± 0.03^Bbc^	6.68 ± 0.18^Abc^	6.31 ± 0.01^Abc^	5.97 ± 0.13^Bd^
30th day	6.49 ± 0.38^Aabc^	6.37 ± 0.03^Ba^	2.54 ± 0.17^Bb^	6.77 ± 0.15^Ab^	6.26 ± 0.02^Ac^	5.83 ± 0.09^Be^
45th day	6.82 ± 0.22^Aab^	6.27 ± 0.34^Ba^	2.58 ± 0.05^Bab^	6.88 ± 0.05^Aab^	6.13 ± 0.16^Ad^	5.58 ± 0.02^Bf^
60th day	6.98 ± 0.36^Aa^	6.02 ± 0.72^Bb^	2.61 ± 0.01^Ba^	7.11 ± 0.09^Aa^	6.03 ± 0.15^Ad^	5.53 ± 0.05^Bf^

^A, B, C^Means shown with different exponential letters in the same row are statistically different (*p* < 0.05)

^a, b, c, d, e^Means shown with different exponential letters in the same column are statistically different (*p* < 0.05)

PCA represents total mesophilic aerobe count; MRSA represents lactobacilli count

### Expression Profile of Stress-Related Genes

The time-dependent variation of the fold changes is shown in Table 6. During ripening, the time-dependent changes of *hsp20, dnaK, groES, ftsH,* and *argH* target genes were not statistically significant (*p* > 0.05).

**Table 6:** Fold changes in expression level of stress-related genes during ripening

Target genes		1st day	3rd day	7th day	15th day	30th day	45th day	60th day	*p* value
*hsp20*	FC	0.808 ± 0.39	0.452 ± 0.13	0.595 ± 0.24	0.780 ± 0.00	0.873 ± 0.28	0.565 ± 0.09	0.834 ± 0.31	0.25
	log_2_(FC)	−0.307	−1.144	−0.746	−0.356	−0.194	−0.821	−0.260	
*dnaK*	FC	0.940 ± 0.16	0.910 ± 0.05	1.904 ± 0.69	1.108 ± 0.20	0.946 ± 0.35	1.027 ± 0.14	1.131 ± 0.63	0.94
	log_2_(FC)	−0.088	−0.134	0.929	0.148	−0.079	0.039	0.178	
*groES*	FC	0.658 ± 0.18	0.827 ± 0.43	0.610 ± 0.12	0.583 ± 0.30	0.507 ± 0.33	0.49 ± 0.18	0.307 ± 0.14	0.27
	log_2_(FC)	−0.602	−0.273	−0.712	−0.776	−0.979	−1.027	−1.699	
*ftsH*	FC	1.169 ± 0.03	0.882 ± 0.11	1.197 ± 0.39	1.315 ± 0.37	1.232 ± 0.33	0.976 ± 0.11	1.132 ± 0.06	0.45
	log_2_(FC)	0.225	−0.179	0.26	0.396	0.302	−0.035	0.18	
*argH*	FC	1.600 ± 0.22	3.201 ± 0.06	2.025 ± 0.12	2.373 ± 0.25	1.995 ± 0.06	1.929 ± 0.06	1.447 ± 0.09	0.12
	log_2_(FC)	0.678	1.678	1.018	1.246	0.996	0.948	0.533	

*FC* fold change

### Whole-Genome Sequencing

**Fig. 1:**
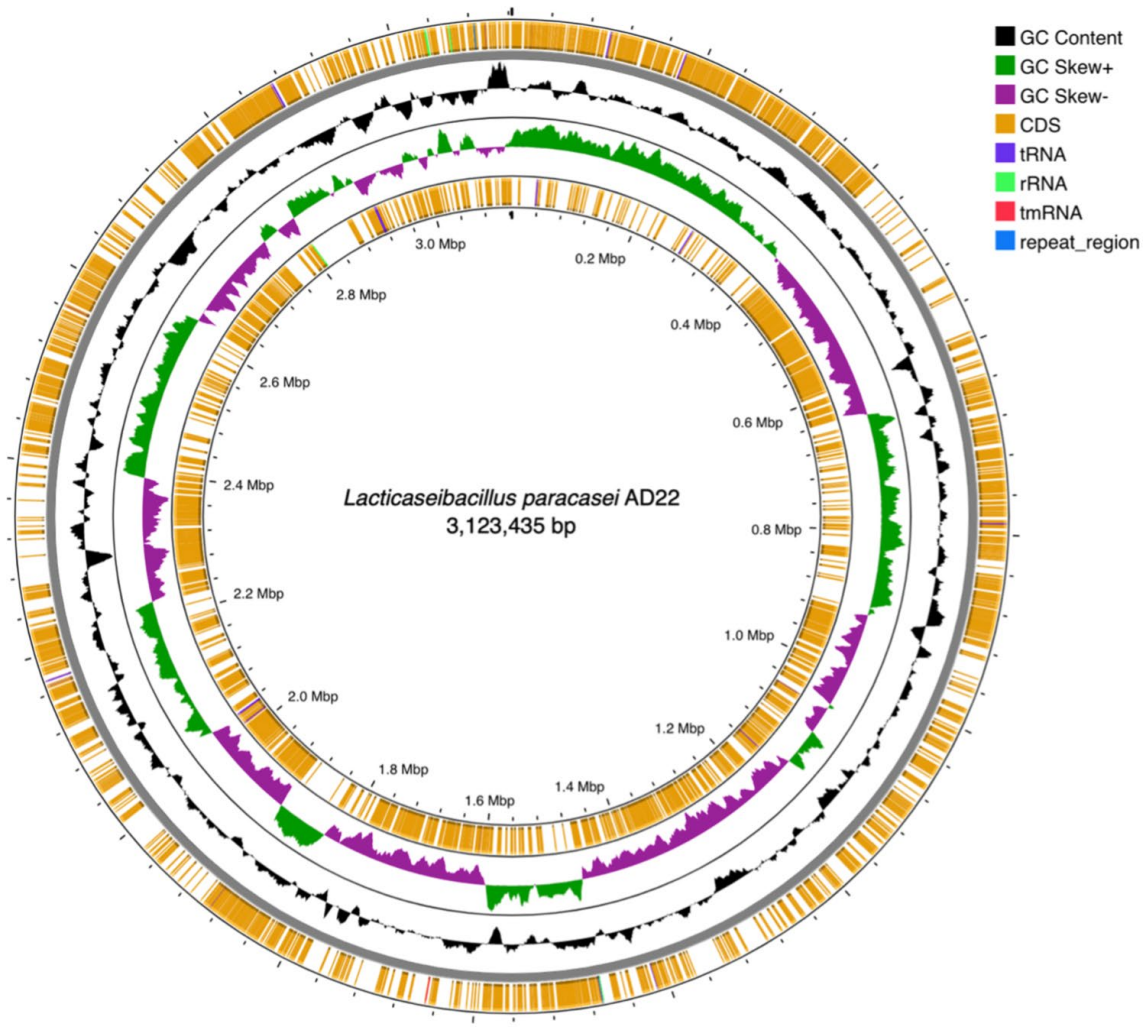
Circular graphical representation of genome annotation distribution. Out-to-in loops: forward CDS regions, GC content, GC skews, reverse CDS regions

**Fig. 2:**
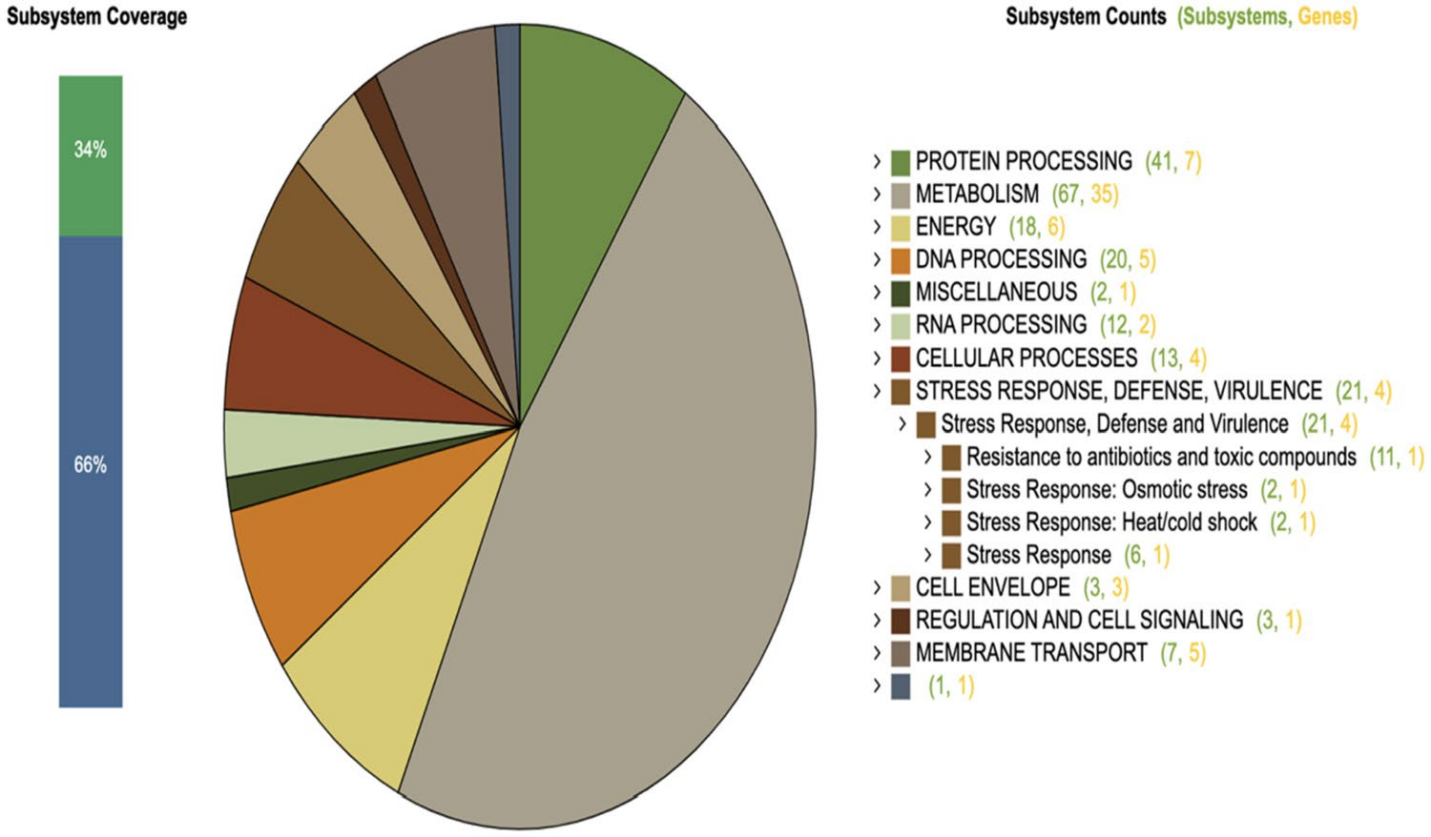
Functional genomic distribution of *Lacticaseibacillus paracasei* AD22 obtained after whole-genome sequencing and assembly

**Fig. 3:**
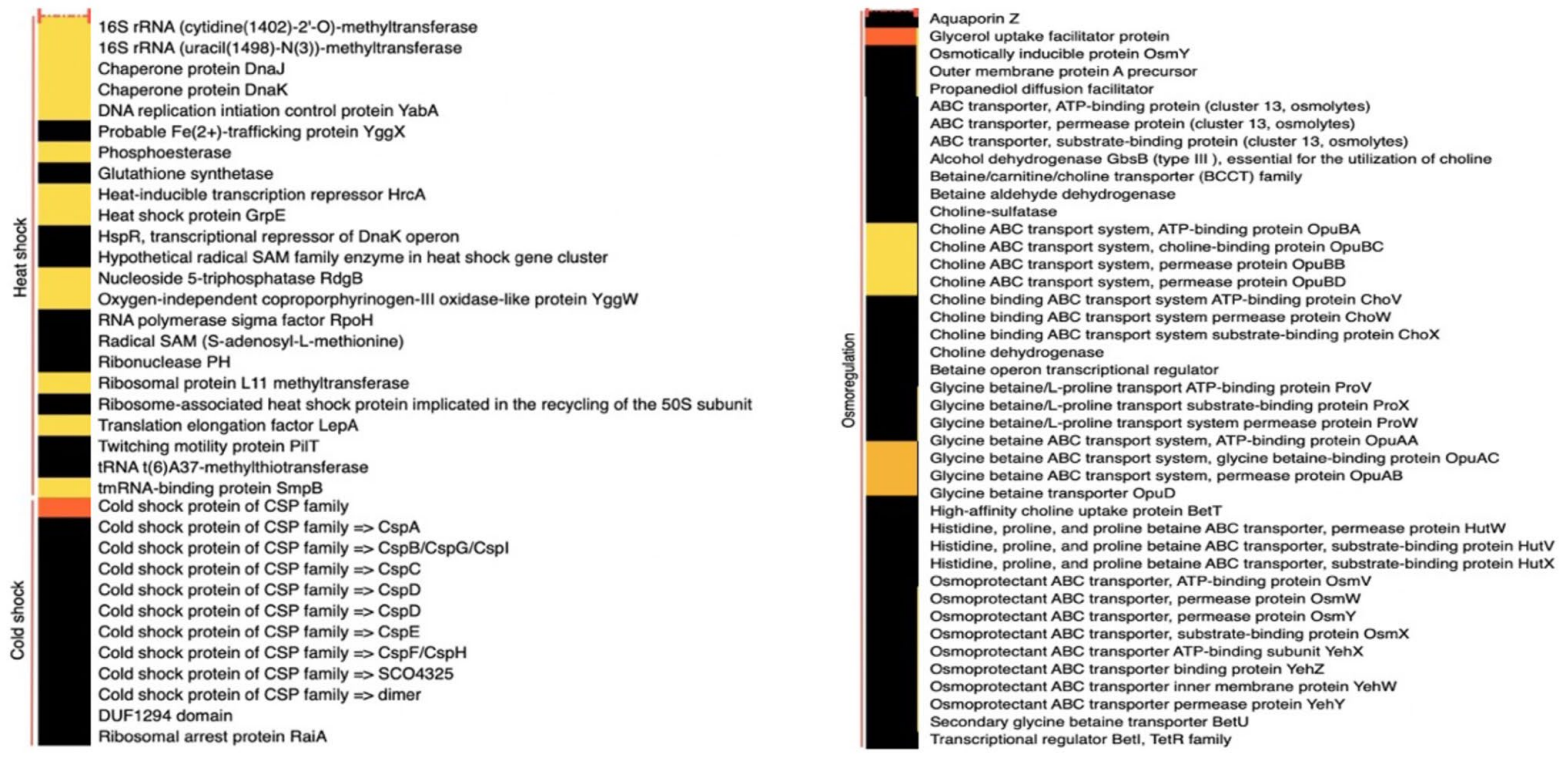
Heat stress and osmotic stress gene presence comparative heatmap with reference genomes (black: no representative protein; bright yellow: one representative protein; dark yellow: two representative proteins; dark orange: three or more representative proteins)

The *Lacticaseibacillus paracasei* AD22 genome sequencing project has been deposited in GenBank under the accession number JAQPCD000000000. This project’s BioProject and BioSample numbers are PRJNA929671 and SAMN32967350, respectively. De novo assembly read results of *L. paracasei* AD22 are indicated in Table 7. Figure 1 illustrates a circular graphic representation of the genome annotation distribution of the strain. Subsystem groups and related gene numbers of subsystems according to the functional genomic distribution obtained after whole-genome sequencing and assembly are shown in Fig. 2. Comparative heat maps of heat-cold stress gene and osmotic stress gene presence with reference genomes are given in Fig. 3. ResFinder4.1 database determined that *L. paracasei* AD22 did not have an antibiotic-resistance gene. VirulenceFinder 2.0 database manifested that *L. paracasei* AD22 did not carry virulence gene. Four regions of interest for bacteriocin production in the *L. paracasei* AD22 genome have been determined. Specifically, these genetic clusters encode the production of Enterolysin A (Class III bacteriocin), carnosine CP52 (class II bacteriocin), Enterocin X beta chain (Class IIc bacteriocin), and the LanT region.

**Table 7:** De novo assembly read results of *Lacticaseibacillus paracasei* AD22

Properties	Values
Average depth*	421.3
Average short reading coverage*	494.5
Min contig coverage threshold*	5.0
Min contig length threshold*	300
Number of contigs above the threshold	165
Number of contigs below the threshold	2138
Longest contig*	639.575
Total length*	3.123.435
GC (guanine/cytosine content, %)	46.22
CDS (protein encoding sequences)	3333
tRNA	56
rRNA	3

^*^bp

## Discussion

### Evaluation of *L. paracasei* AD22 in Terms of In Vitro Physiological and Safety Traits

To qualify as a probiotic, the potential candidate must possess certain functional and safety characteristics, including the stomach (low pH, gastric juice, and pepsin) and intestines (pancreatin and bile salts) conditions, adhesion capacity, hemolytic activity, and sensitivity to antibiotics [20, 36]. *L. paracasei* AD22 showed good probiotic properties, showing remarkable growth in the acidic pH 2 milieu. Kumar et al. and M’hamed et al. reported that no growth occurred due to the incubation of isolated lactobacilli at pH 2 [37, 38]. Adaptation to bile salts is affected by changes in carbohydrate fermentation and glycosidase activity [39], exopolysaccharide production [40], the composition of membrane proteins and fatty acids [41], and human intestinal mucosa and the inhibition of the attachment of pathogens [42]. Like the survival of *L. paracasei* AD22, Xu et al. stated that *L. paracasei* L1 strain exhibited 99.8%, 99.2%, and 98.3% tolerance to various levels of exposure to bile salts [20]. Tolerance to pepsin and pancreatic enzymes is also a critical factor in the survival of probiotics after entry into the host [19]. Lu et al. found the survival rate of *L. paracasei* X11 strain in the simulated gastric and intestinal environment close to the current study [43], while Yilmaz et al. reported the survival rate of *L. paracasei* KS-199 as 64.1%. *L. paracasei* AD22 showed tolerance to all NaCl concentrations tested, indicating that the strain withstands the adverse effects of high osmotic pressure in the high salt environment of the gastrointestinal tract and maintains osmotic pressure balance [44].

Adhesion of probiotics is a phenomenon that begins with contact with host enterocytes and continues through various surface interactions [45]. Adhesive strains exhibit an elevated level of hydrophobicity, and the degree of adhesion depends on the surface potential [46]. *L. paracasei* AD22 is thought to have specific cell surface molecules that play a role in its adhesion ability. While autoaggregation appears to be associated with attachment to epithelial cells [47], coaggregation exhibits antipathogenic traits by forming a defensive barrier against the colonization of pathogenic microorganisms [48]. Coaggregation properties have been found to control a microenvironment around pathogens of probiotic strains and increase the concentration of antimicrobial substances released during aggregation [49]. Similar to the current study, Xu et al. found that the surface hydrophobicity of *L. paracasei* L1 was 38.1%, and its autoaggregation ability increased significantly with incubation time [20] Xu et al. stated that *L. paracasei* ATCC 25598 could coaggregate against *L. monocytogenes* (38.7%) and *S*. Typhimurium (44.7%) [50]. Amini et al. found the coaggregation level of *L. paracasei* AS20 to *L. monocytogenes* to be 56.7% at the end of the 24th hour [51]. Breyer et al. reported the coaggregation percentages of *L. paracasei* LB 6.4 to *E. coli* ATCC 25922 and *S. aureus* ATCC 25923 as 56.3 and 65.5, respectively [52]. According to the findings of our study, *L. paracasei* AD22 is thought to be beneficial for the competitive elimination of enteropathogens. Caco2 and HT-29 cell lines are the most widely used cell lines for evaluating bacterial strains’ adhesion and anti-adhesion properties [53]. Krausova et al. stated that the adhesion level of *L. paracasei* E3TA to Caco2 cells was ~ 35% [53]. Unlike *L. paracasei* strains studied by Xu et al., Xu et al. and Breyer et al., *L. paracasei* AD22 has a high adhesion ability to Caco2 cell lines [20, 50, 52]. Yasmin et al. stated that cell surface properties still need to be studied in vivo to understand the interaction within the host cell better [18].

*L. paracasei* AD22 does not show hemolytic activity. Regarding the safety of probiotics, lack of hemolytic activity is essential in selecting probiotic strains, as virulence is not observed among hemolysin-deficient bacterial strains [54]. It has been demonstrated that *L. paracasei* AD22 exhibits proteolytic activity. The hydrolysis site produced in skimmed milk agar may be related to the amount of protease produced by the organism. Proteolytic enzymes are a group of enzymes that catalyze many different biochemical reactions and can be used in many industries. In particular, in the dairy industry, the proteolytic system of lactic acid bacteria provides essential amino acids to cells during their growth, is involved in the utilization of casein, and plays a remarkable role in regulating the organoleptic properties of fermented milk products [55]. *L. paracasei* AD22 showed resistance to vancomycin, streptomycin, and ciprofloxacin while showing sensitivity to other antibiotics tested. Anisimova et al. and Liu et al. reported that all lactobacilli tested showed intrinsic resistance to streptomycin, kanamycin, vancomycin, and ciprofloxacin [19, 56]. The resistance of lactobacilli to antibiotics such as aminoglycosides and the absence of electron transfer enzymes mediated by the cytochrome responsible for the metabolism of the antibiotic cannot be taken into the cell, and this is considered as natural resistance [57]. Resistance to vancomycin has been attributed to the presence of D-Ala-D-lactate in its peptidoglycans instead of D-Ala-D-Ala, which is the target of the antibiotic [57]. The European Food Safety Authority (EFSA, 2012) considered the evaluation of vancomycin on *L. paracasei* as ‘not required’ in the guideline on the antimicrobial susceptibility assessment [58]. Resistance to ciprofloxacin is thought to be due to natural features such as the impermeability of the cell wall or the flow mechanism. Natural resistance to antibiotics is not seen as a threat to the health of animals or humans [59]. One of the crucial mechanisms of probiotics’ action is their antagonistic effect against microbial pathogens with their antimicrobial metabolites. Our study findings comply with Miao et al., Cizeikiene and Jagelaviciute, and Qureshi and Li [60–62]. The main compounds responsible for the antimicrobial activity of CFS are lactic and acetic acids, long-chain fatty acids and esters, and proteinaceous compounds [63].

### Evaluation of *L. paracasei* AD22 in Cheese Matrix

It is critical for white-brined cheese to maintain a concentration level of 10^7^ cfu/g for probiotics [64]. It was observed that *L. paracasei* AD22 achieved this criterion by reaching 7.11 log. *Lactobacillus* growth was observed on MRS agar plates in the control group. Non-starter LAB can be found in cheese by maintaining its viability from milk pasteurization or post-pasteurization contamination in the dairy [65]. Leeuwendaal et al. reported that the cultures used as adjuncts with probiotic properties continued at 10^7^–10^8^ cfu/g levels in cheese during ripening [66]. Buriti et al. emphasized that the lactobacilli count in Minas cheese produced with commercial starter culture and *L. paracasei* increased from 6.66 to 8.44 log cfu/g in 21 days and that protocooperation/symbiosis might have occurred between cultures [67]. The differences detected between cheeses in the study were attributed to the initiating microorganism rather than *L. paracasei*. When the total mesophilic aerobe counts were examined, there were differences from the first day between the control group and the cheese with added *L. paracasei* AD22. However, the total aerobic mesophilic microorganism count was suppressed in *L. paracasei* AD22 added cheese after day 45. Similar to the present study, Buriti et al. concluded that the pH value of cheese with probiotics was lower than the control group. Bruti et al. reported that using additional cultures, such as *Lactobacillus paracasei*, for producing fresh Minas cheese can potentially improve the health properties and quality of the product [67]. However, adding probiotic microorganisms and lactic cultures can solve the problem of weak acidification.

### Evaluation of Stress Responses in Cheese Matrix of *L. paracasei* AD22

In the current study, *L. paracasei* AD22 was kept at 4 °C during the ripening of brined white cheese and was exposed to a brine environment containing 12% salt and a pH change. Examining the *argH, ftsH, hsp20, dnaK,* and *groES* genes was deemed appropriate because the main stress responses result from cold shock, osmotic, and acidity changes. The present study determined the downregulation of genes encoding groES chaperone proteins and hsp20 at refrigerator temperature. The fold changes of these genes during ripening were not statistically significant. However, the stress response of the *dnaK* shifted to upregulation, which is thought to be due to the cross-protection mechanism during ripening. Liu et al. found that *dnaK* as a cold stress response is significantly downregulated due to complex translational rearrangements in *L. plantarum* [68]. Duru evaluated the expression of heat shock response genes such as chaperone genes, *Clp* protease genes, and the chaperone-binding gene *grpE* [69]. All these genes have been reported to be downregulated in cold environments in *Leuconostoc gelidum*, *Lactococcus piscium*, and *Lactobacillus oligofermentans*. GroES/GroEL and DnaK/DnaJ have been reported to be induced during environmental conditions such as osmotic and saline stresses, oxidative stress, pH extremes, UV radiation, and the presence of toxic compounds, as well as heat shock stress [70–73]. Adu et al. emphasized that the Hsp20 family heat shock protein is highly upregulated in response to heat stress compared to other molecular chaperones and that this protein can serve as a valuable biomarker for *L. casei* GCRL163 in revealing long-term heat stress [74]. *ftsH* is regulated under the osmotic stress response and heat shock cross-resistance [75]. Expression of *ftsH* in *Oenococcus oeni* increased with elevated temperatures and osmotic shock. Bagon et al. emphasized that *ftsH* is involved in tolerance of *L. plantarum* WCFS1 and promotes bacterial survival, thus increasing survival under elevated levels of biliary stress [76]. *argG* and *argH* are critical genes involved in aspartate and arginine metabolism [77]. Breyer et al. emphasized that overexpression of *atpD* and *argH* genes is related to the response to acid and maintaining the presence of LAB in this environment [52]. Huang et al. reported that the expression of *argH* and *argJ* in the arginine biosynthesis pathway of *Streptococcus thermophilus* T1C2 was almost fourfold upregulated compared to control, indicating that *S. thermophilus* T1C2 began to synthesize arginine to meet growth or survival requirements [78]. In our study, the *argH* gene for the *L. paracasei* AD22 showed an upregulation profile over time during cheese ripening. The difference between the fold changes over time was not significant. However, it is thought that *L. paracasei* AD22, resistant to pH 2.5 in MRS broth, will be a suitable probiotic candidate in cases where the pH drop is high in industrial use.

### Whole-Genome Sequencing of *L. paracasei* AD22

The suitability of the candidate species for use as probiotics is supported by their lack of antibiotic resistance and virulence factors and their ability to produce bacteriocins [79]. No resistance gene was found for *L. paracasei* AD22. Likewise, Tarrah et al. stated that there are no potential negative features such as antibiotic resistance and virulence factors for *L. paracasei* DTA93 and reported that it can be evaluated in food products within the scope of health-promoting cultures [80]. The most functional part of the genomic distribution of *L. paracasei* AD22, showing its potential biological functions, consists of metabolism, protein processing, and stress response functions. Genomic analysis provides in-depth information on the functional mechanisms involved in potential probiotics and their adaptation to the environment. Zheng et al. reported that the immune system process, adhesion, amino acid, and carbohydrate metabolism constitute the majority of the functional genomic features of *L. rhamnosus* CY12 and stated that the strain has metabolic ability and tolerance [15]. Comparative heat maps with reference genomes identified genes conferring heat and cold shock tolerance, such as heat shock proteins and members of the cold shock protein family. Accordingly, *L. paracasei* AD22 encodes the DnaK and DnaJ chaperone system that helps protein folding under stress conditions. The protein repair function of DnaK, GrpE, and especially DnaJ is likely to be part of the role of these proteins in regulating the heat shock response [81]. The gene *yabA*, which encodes DNA replication-initiating protein, has a functional relationship with the replication mechanism [82]. While HSPs are essential for cell development, their catalytic activity becomes particularly important under stress conditions that lead to the accumulation of unfolded or misfolded proteins. These include genes encoding the chaperone protein (DnaK, DnaJ) and the nucleoside 5′-triphoaphate RdgB, essential in adaptations to psychrophilic lifestyles [83]. LepA induces back-translocation of tRNAs on the ribosome [84, 85], which is formed by progressing from the post-translocation complex to the pre-translocation complex [86]. A thorough understanding of the cold-adaptation process can be used to optimize low-temperature fermentations. It can provide insight into methods of controlling the growth of pathogenic bacteria and spoilage, which affect the shelf life and safety of foods stored in the refrigerator [87]. On the other hand, cold shock refers to the reaction of mesophilic or psychrotrophic microorganisms to a sudden and significant drop in temperature. Protein-encoding genes facilitating glycerol uptake are necessary for the intracellular transport of glycerol and metabolism functions in bacteria [88]. When osmotic stress responses were examined, it was concluded that *L. paracasei* AD22 contains opuBA-opuBB-opuBC-opuBD operons that are involved in the transport of osmoprotectants such as glycine betaine, proline, choline, or carnitine [89]. The expression of opuB operons is regulated in response to the increased osmolality of the growth medium [90]. Kiousi et al. stated that *L. paracasei* SP5 contains genes necessary to produce glycine betaine binding factors and osmolyte transporters that accumulate in the cell under hypertonic shock [91]. In addition, it has been reported that the synergistic activity of GrpE with DnaK and DnaJ during hyperosmotic shock reduces the damage to the macromolecular mechanism of the cell. The BAGEL4 analysis revealed the existence of Enterolysin A, Carnosine CP52, Enterocin X beta chain interested in bacteriocin production in the *L. paracasei* AD22 genome. In addition, the lantibiotic transporter LanT region identified in its genome can be evidence of the possible existence of new clusters for lantibiotic production [92].

In conclusion, *L. paracasei* AD22 can be used in developing new functional products and producing fermented foods since it has the structural features that a probiotic culture should have. Considering *L. paracasei* AD22 more comprehensively within the framework of omics approaches will enable it to be evaluated in terms of revealing the characteristics and capabilities of the strain, obtaining scientific data regarding health-promoting studies.

## Data Availability

The datasets used and analyzed during the current study are available from the corresponding author on reasonable request.
